# Young people in iNaturalist: a blended learning framework for biodiversity monitoring

**DOI:** 10.1080/21548455.2023.2217472

**Published:** 2023-06-01

**Authors:** Christothea Herodotou, Nashwa Ismail, Ana I. Benavides Lahnstein, Maria Aristeidou, Alison N. Young, Rebecca F. Johnson, Lila M. Higgins, Maryam Ghadiri Khanaposhtani, Lucy D. Robinson, Heidi L. Ballard

**Affiliations:** a Institute of Educational Technology, The Open University, Milton Keynes, UK; b School of Education, Durham University, Durham, England; c Natural History Museum, London, UK; d California Academy of Sciences, San Francisco, CA, USA; e Natural History Museum Los Angeles, Los Angeles, CA, USA; f UC Davis, Davis, CA, USA

**Keywords:** Community citizen science, blended learning, young people, biodiversity monitoring

## Abstract

Participation in authentic research in the field and online through Community and Citizen Science (CCS) has shown to bring learning benefits to volunteers. In online CCS, available platforms present distinct features, ranging from scaffolding the process of data collection, to supporting data analysis and enabling volunteers to initiate their own studies. What is yet not well understood is how best to design CCS programmes that are educational, inclusive, and accessible by diverse volunteers, including young people and those with limited prior science experiences who are rather few in CCS. In this study, we interviewed 31 young people, aged 7–20 years old, who used iNaturalist, an online biodiversity monitoring platform, and identified how different forms of participation online and in the field facilitated (or inhibited) certain forms of learning, as defined by the Environmental Science Agency framework. Findings revealed that iNaturalist enabled participation of young people including those with limited science experiences and facilitated science learning such as the development of science competence and understanding. A blended learning framework for biodiversity monitoring in CCS is presented as a means to support the development of hybrid, educational, and inclusive CCS programmes for young people.

## Introduction

Community and Citizen Science (CCS) refers to a diverse set of participatory approaches to authentic research, ranging from collecting and analysing data for scientists to defining the focus and methodology of a study in community-led research (Herodotou et al., 2017; Ballard et al., 2017; Herodotou et al., 2021a). An increasing body of studies has documented the positive impact online CCS participation has on science learning for volunteers such as enhanced science knowledge, development of scientific skills such as data collection and analysis, scientific literacy, as well as broader learning outcomes such as greater awareness and personal change (e.g. Herodotou et al., 2022; Aristeidou & Herodotou, 2020; Jennett et al., 2016). These learning benefits are rather accessed and enjoyed by a specific cohort of volunteers: educated, white, middle-aged, middle to upper class individuals (e.g. Blake et al., 2020; National Academies of sciences, Engineering, and Medicine, 2018). Young volunteers with limited science experiences as well as historically underrepresented groups, are less likely to participate in CCS (Blake et al., 2020; Fiske et al., 2019). Having a different sense of autonomy, adults are more likely to participate in more and diverse stages of the scientific process, such as data analysis while they are found to have a set interest in specific CCS topics because of personal experience, training, or previous education (Curtis et al., 2018; Sharma et al., 2019). Yet, it is noted that participation in more stages of the scientific process may not necessarily result in superior learning. Four field-based studies related to an urban bat ecology project (Greving et al., 2022) identified that topic knowledge and science-related attitudes improved similarly in both the group who took part in data collection and the group who both collected and analysed data. Considering differences between adults and young people, distinct forms of scaffolding, such as training, may be needed for different groups of volunteers, that are tailored to their previous experiences and age capacities as well as their time availability and commitment (Echeverria et al., 2021; Flagg, 2016; Frensley et al., 2017).

A rather limited body of research has examined the participation of young volunteers in CCS, with evidence pointing to, for example, the development of environmental science agency, after youth took part in data collection processes and were given opportunities to disseminate their findings (Ballard et al., 2017). Yet, this was not the case for all youth; learning benefits were moderated by factors such as youth not understanding collected data and how a CCS task was facilitated and presented (Harris & Ballard, 2021). Also, little is known about how the design of online CCS platforms enables or inhibits certain learning outcomes (Herodotou et al., 2022). Online CCS platforms present unique design features and, therefore, allow for different types of participation and associated learning experiences by volunteers. For example, Zooniverse (zooniverse.org) is mainly supporting processes of data analysis by asking volunteers to engage with pre-collected datasets; nQuire (nquire.org.uk) enables volunteers to design, pilot, manage and run their own scientific studies; and iNaturalist (inaturalist.org) scaffolds the process of data collection and species identification. In this study, we detail how young people take part and learn from blended learning CCS activities supported by the iNaturalist platform, a biodiversity monitoring platform focusing on species’ identification.

### Types of participation in CCS

Participation in CCS projects has been described in terms of the possible tasks a volunteer may be engaged with including defining a research question, gathering information, developing a hypothesis, designing a study, collecting data, analysing samples and data, interpreting data, drawing conclusions, disseminating findings, and discussing results (Wiggins & Crowston, 2012).

Regarding field-based and short-term CCS, the analysis of ethnographic field notes from 81 youth participating in 15 bioblitz events organised by Natural History Museums in the UK and the US (Lorke et al., 2021) demonstrated five types of participation as follows: (1) *Exploring* referring to young people's effort to explore and search for wildlife, (2) *Observing* referring to young people using their senses to find, watch, listen, notice organisms, (3) *Identifying* referring to young people adding a name to an organism with the help of more knowledgeable others, using their prior knowledge, or getting help from iNaturalist (automatic recommendations), (4) *Documenting* referring to the creation of a written or digital record of a species (e.g. photo, written notes), (5) *Recording* referring to sharing this record with scientists either by using iNaturalist or by sharing physical records with programme facilitators at a field-based CCS event for use in research.

Regarding online CCS, a working framework mapping types of participation and youth science learning (Herodotou et al., 2022), identified three main types of participation observed within and out of the online platform, Zooniverse, that were shown to enable learning: (a) *doing a task:* mainly analysing data scientists shared such as counting penguins on an image, (b) *exploring*: searching and reading about projects on Zooniverse or searching for information online or offline, and (c) *communicating:* either via the forum on Zooniverse or with more knowledgeable others to receive help and complete a task.

The types of participation presented in this paper (i.e. RQ2) elaborate on the work of Lorke et al. (2021) by (a) presenting *Documenting* and *Recording* as a joint type of participation; *Documenting* and *Recording* in iNaturalist were found to be linked processes observed when uploading an observation to the platform, and (b) identifying and proposing a new type of participation coined as *Communicating*.

### Youth participation and learning in CCS

Informal science experiences referring to young people’s participation in out-of-the-classroom activities, at home, online and at institutions such as museums, zoos and science clubs have shown to influence interest and learning in science (Bell et al., 2009). Youth participation in informal science activities has shown to vary with some young people taking part frequently in designed and community-based activities (museums, science centres, clubs, etc), others taking part only in specific science practices, and others participating across a range of science practices. Many young people from minority communities, while interested in science, were found not to take part in designed and community activities. On the contrary, those from socially advantageous backgrounds were found to take part in such activities despite a low interest (Godec et al., 2022). Specific types of participation were shown to be more accessible than others such as everyday learning experiences (watching science programmes, talking to others about science, etc) as opposed to participation in designed science spaces and school-led enrichment (e.g. school science trips), necessitating interventions that mitigate observed inequalities in access and participation (DeWitt & Archer, 2017). Amongst the strategies proposed to widen participation has been the design of science projects that are relevant to the interests of youth, improvements in the accessibility of science experiences and appropriate support through mentoring and role model connections (Vogt et al., 2016).

An emerging body of studies shows learning benefits from taking part in online CCS activities, not only for adults, but also for young volunteers. Examining the participation of 183 young people aged 5–19 on iNaturalist, Aristeidou et al. (2021a) showed that the more days young people used on iNaturalist, the more their daily contributions were and the more systematic visitors they became. In a follow up study with 249 young volunteers, Aristeidou et al. (2021b) identified that the observations of young volunteers were contributing to systematically mapping biodiversity, supporting relevant science and research, as they included information such as location, time/date and photo needed for the iNaturalist community to verify their identifications. Volunteers’ observations on iNaturalist are focused on plants or insects and volunteers rarely observe the same species twice (Di Cecco et al., 2021).

An intervention with two design studies engaging people in citizen-led inquiries, via the nQuire platform, indicated that participants gained content knowledge and practised science skills, such as observation and identification, data collection and annotation (Aristeidou et al., 2020). Further, they developed transferable skills, such as digital literacy, writing and self-efficacy. The level of participation in CCS activities and type of learning were affected by the different scaffolding levels that the platform offered across the two studies such as guidance through an inquiry learning framework and social interaction with a moderator facilitating discussions and encouraging participation. Another study engaging young people with citizen-led inquiries on nQuire stressed the importance of mobile-friendly platform design to promote learning and participation (Aristeidou & Herodotou, 2018). A survey with 150 nQuire adult participants noted learning benefits from participation in online CCS studies (led by scientists) including awareness and behavioural change such as actions to provide a habitat for pollinators, yet challenges were noted when volunteers were asked whether they would create their own studies (Herodotou et al., 2021a ), raising the need for further support should CCS participation include ‘extreme citizen science’ forms (Haklay, 2013).

Specific online types of participation (Herodotou et al., 2022) were shown to enable specific forms of learning, for example, communicating with more knowledgeable others was shown to relate to a desire to become a scientist, whereas doing a task was related to the development of scientific skills and expertise in using tools. Other studies with young people from Zooniverse showed that young people are rather ‘visiting’ Zooniverse as their participation is not systematic (Herodotou et al., 2020) and those more likely to report learning benefits from participation are those with significant prior experiences with science (Herodotou et al., 2021b).

### iNaturalist: A CCS platform supporting blended learning

iNaturalist is an all-taxa platform, both a website and an app, that allows participants to: (1) contribute observations of organisms or evidence of an organism (e.g. tracks, feathers, shells); (2) get help with identifications of those organisms; (3) keep track of species they have seen; (4) join and create projects within the platform; (5) explore the global set of all users’ observations; and (6) interact with other users around those observations (through providing identifications and/or leaving comments).

While iNaturalist participants can submit observations without any documentation of an organism, or of organisms that are not wild, *verifiable* observations include either a photograph or an audio recording of a wild organism, along with associated spatiotemporal metadata. Verifiable observations are then identified to the finest possible taxonomic resolution via a combination of computer vision algorithm and the iNaturalist community. Once a verifiable observation has two or more suggested identifications and more than two-thirds of the identifications agree at a species level, the observation is deemed as ‘Research Grade’ (see www.inaturalist.org/posts/39072-research-grade). Research Grade observations are exported to other species occurrence databases, including the Global Biodiversity Information Facility (GBIF). iNaturalist is used extensively as the data-collection platform for many CCS programmes, especially those focused on which species are found where and when. iNaturalist is often used in bioblitzes, where one of the goals is to understand what species occur in a specific place, like a park or a city. It is the primary platform for the largest global CCS bioblitz, City Nature Challenge (www.citynaturechallenge.org).

**Figure 1.**
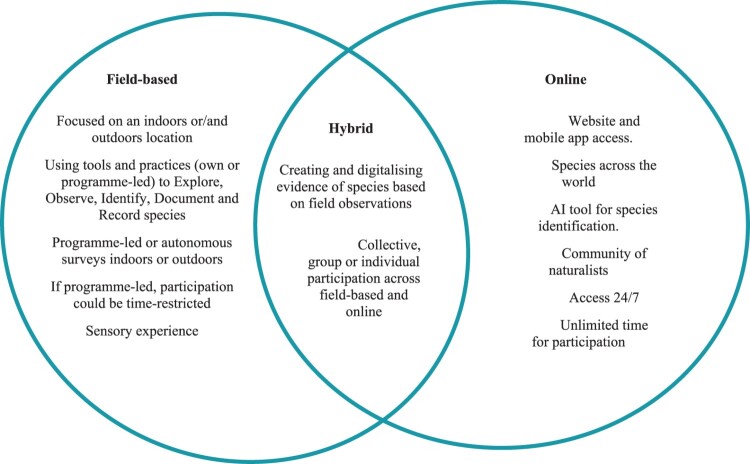
Design features supporting hybrid participation and blended learning in iNaturalist.

iNaturalist supports blended forms of learning and participation in both online and field-based contexts (Figure 1). Blended learning describes ‘all types of education that include some aspect of face-to-face learning and online learning’ (Hrastinski, 2019, p. 1). There are several definitions and models of blended learning, especially discussed and applied in formal education, in which the prevalence of online versus face-to-face or offline components varies depending on the implementation and learning objectives. Blended learning has shown to be more effective than online only learning in education (e.g. Topping et al., 2022) impacting outcomes such as academic performance, self-regulation, satisfaction, and engagement (Ashraf et al., 2021).

Blended learning experiences have also been developed in informal settings, for example, the use of augmented reality in museum education (Lee et al., 2021). In CCS, the use of information and communication technologies (ICT) has provided new opportunities for data collection at scale and reduced geographic barriers to citizens’ participation. In particular, mobile devices such as phones and camera traps, and web/mobile applications have been used to observe and record environmental data about phenomena such as climate change, heatwaves, and biodiversity (Herodotou et al., 2021a; Green et al., 2020; Wallace & Bodzin, 2017). Such implementations were shown to have a positive impact on attitudes towards CCS and STEM careers (Wallace & Bodzin, 2017), maintain engagement in long-term monitoring projects, especially when an app is well supported, fun and free (Lee & Nel, 2020) and promote data quality in citizen-led CCS (Herodotou et al., 2021a). In this paper, we studied iNaturalist as a blended learning CCS environment. Offline components involve outdoor or indoor settings where organisms inhabit, colonise or transit and in which iNaturalist users engage in ‘exploring, observing, identifying, recording and documenting biodiversity’ (Lorke et al., 2021). Online components are accessed through the iNaturalist mobile app and online website and mainly relate to identifying and documenting species with the help of an online community and the AI identification system embedded in iNaturalist.

Amongst the challenges in blended learning is the limited flexibility learners may have in defining the ‘blend’, and social interaction usually taking place offline while monitoring learning online (Boelens et al., 2017). It could be argued that, in iNaturalist there is considerable flexibility for young people to define the ‘blend’, as youth are self-regulating their engagement with both the iNaturalist mobile app and/or website and the natural environment. For instance, they can use iNaturalist to record and document nature in their neighbourhood or use iNaturalist in organised CCS events such as Bio-blitzes, determining the offline components of the ‘blend’. Similarly, they can decide on how to use the iNaturalist online affordances such as when to upload and identify a photo and search for observations of others. It could be argued that learning is self-regulated as volunteers set up and pursue their own goals by regulating their emotions, thoughts, and actions (Zimmerman, 2000) while engaging with iNaturalist, the indoors or outdoors space, and perhaps the broader goals of a CCS programme. In terms of social interaction, this is supported in both online and offline settings. In offline settings, this can be achieved through participation in field-based CCS events where organisers debrief a CCS event or scaffold the process of data collection, or online via iNaturalist features such as identifying and commenting on observations of others. Monitoring of learning is not an explicit objective of iNaturalist, yet this could be achieved through the extraction of log files, used in other studies (Herodotou et al., 2022) for understanding how youth's participation relates to learning outcomes, suggesting that the online dimension of the ‘blend’ can provide valuable information about learning and participation through the interaction traces of participants.

### Aim and research questions

The aim of this study was to understand how the design of a biodiversity monitoring CCS platform, iNaturalist, supports learning for young people. We interviewed 31 young people aged 7–20 years old who took part in either a CCS programme or used iNaturalist on their own. In addition, for three of these participants (hereafter called ‘focal cases’), we complemented our understanding of their learning and participation with data sources collected in the field during and after they took part in a CCS programme coordinated by a Natural History Museum. We answered the following Research Questions (RQs):

RQ1: What science learning outcomes (as captured by the framework of Environmental Science Agency, see Section 2) do young people report after engaging with a biodiversity monitoring platform?

RQ2: How do different types of participation in a biodiversity monitoring platform enable or hinder science learning outcomes?

RQ3: How do previous science experiences of young people relate to science learning outcomes?

This examination forms part of a broader research grant funded by the National Science Foundation, Wellcome and ESRC that identified how young people take part and learn from participation in museum-led online and field-based CCS programmes and offered recommendations as to how CCS could become more accessible to diverse youth. This was achieved through an interdisciplinary collaboration between three universities: UC Davis, The Open University, and the University of Oxford, and three museums: Natural History Museums in London and Los Angeles and the California Academy of Sciences. Young people's participation in online settings was examined in two CSS platforms; in this paper, we report on findings from the iNaturalist platform only.

## Theoretical framework: Environmental Science Agency

The framework of the Environmental Science Agency (ESA) informed our understanding of youth learning and participation in CCS (Ballard et al., 2017). ESA guided the design of interview questions, the codebook used for analysing data and its interpretations. The development of ESA has been influenced by democratic science pedagogy researchers Basu and Calabrese Barton (2010) and the socio-cultural theorists Lave and Wenger (1991). In ESA, learning is understood in relation to three dimensions: (a) ESA1: youth understanding environmental science content, science practice, and science norms, (b) ESA2: identifying own expertise within environmental science, and (c) ESA3: using science experiences to bring change for themselves and their communities. The development of ESA has similarities to how youth develop practice-linked identities (Nasir & Hand, 2008) and agency with science (Calabrese Barton & Tan, 2010) and is influenced by factors such as the context of participation in learning settings and the features of the learning settings themselves.

## Methodology

Study participants were 31 young people (aged 7–20 years old) who had used the iNaturalist platform. They were either participants of short-term or long-term field-based CCS events or programmes coordinated by Natural History Museums in the US and the UK or used iNaturalist on their own to make nature observations. They were recruited through an announcement shared in social media inviting volunteers to take part in interviews for the Learn Cit Sci (Learning & Environmental Science Agency Research Network for Citizen Science) project. Participants or their guardians (if younger than 16 years old) consented to take part in the study by completing an online form. Ethical clearance was gained by The Open University UK prior to approaching participants. Out of the 31 participants, 12 were female and 19 were male. Eight participants were 7–10 years old, 13 participants were 11–16 years old, and ten participants were 17–20 years old. Seventeen participants were in the US, four in the UK, two in Canada, one in Costa Rica, and seven did not disclose their location. Twelve of them were sharing their account with other family members.

### Methods of data collection

To address the RQs of this paper, we collected (a) qualitative interview data from 31 young participants of the iNaturalist platform and (b) additional data from three of the 31, or three focal youth (pseuds. Daniel, Andrea, Carol) of similar age (11–12) and location (LA, California, U.S.A.), combining online and field-based datasets (Table 1). These focal youth were the only young people that agreed to participate in both the field-based and the online research programme of Learn Cit Sci. Their voluntary participation in both research programmes was a key selection criterion to study the interaction and influence of field-based activities and the virtual platform in the context of each research question. These participants took part in a CCS programme coordinated by a natural history museum, which records urban species occurrence over a year in local areas using iNaturalist. The programme offers training to parents/guardians to support their children’s participation and museum events where participants can meet and interact with museum experts. These focal youth illuminated the blended learning aspects of participation and learning, which are presented through three individual snapshots or vignettes, each one specifically addressing one of the three research questions. Multiple data sources for these three focal youth (see Table 1), combined using a convergent parallel design (Edmonds & Kennedy, 2016) offered a complementary understanding of science learning and participation in a CCS blended environment through iNaturalist. As shown in Table 1, the chosen methods of data collection sought to collect data and answer all three RQs simultaneously, thus serving triangulation purposes.

**Table 1.** Data collection methods.

Data collection methods	Information gathered by data collection methods
**1.Online research participants** (*N* = 31) 1.1 Semi-structured interviews online via Skype (audio recorded, ∼40 min)	1.1 Elaborated on how youth used iNaturalist, their interactions with the physical space and nature, their previous science experience and interest, self-perceptions of learning through iNaturalist, and captured the potential influence of design features of iNaturalist.
**2. Focal research participants** (*n* = 3) Instrument 1.1 in addition to: 2.1 Paper-based pre-survey (Prior to field-based activities) 2.2 Paper-based post-survey (post to field-based activities) 2.3 In person semi-structured interview (audio recorded, ∼40 min) 2.4 On-site observation fieldnotes	2.1 Gathered information such as prior engagement with science activities and attitudes towards science and monitoring nature. 2.2 Gathered recollections of their field activities, learning perceptions and new competence in the research field-based activities to monitor biodiversity with iNaturalist. 2.3 Elaborated on aspects covered by 2.1 and 2.2 with an emphasis on field-based activity, self-perceptions of learning, and influence of event/programme features on learning. 2.4 Using an observation protocol, field researchers documented 20 min intervals of individual youth field-based activity in two to four sessions lasting approx. 1 h each. These focused on capturing youth’s scientific thinking and practices, evidence of environmental agency while monitoring nature and using or attempting to use iNaturalist.

During the interviews, the guardians of participants younger than 16 years old were asked to listen to the interview, yet remain in the background avoiding any disruption. Example interview questions were: (a) (RQ1): Do you think by doing activities on iNaturalist, you got better at learning science or doing science? (b) (RQ2): What do you like about doing when using iNaturalist? (c) (RQ3): How did your previous experience help you to use iNaturalist?

### Methods of data analysis

#### Online participants

Interview data were analysed following principles of thematic analysis (Braun & Clarke, 2006; Broadbent et al., 2020). A predefined code-book developed to address the objectives of the Learn Cit Sci project was used to capture youth participation and learning in blended settings (field-based and on iNaturalist). The codebook has been iteratively developed, reviewed, and refined by the project team through five cycles of iteration (See Campbell et al., 2013). We uploaded field note observations and interview transcripts to Dedoose (Version 8.0.45) and survey data were entered in spreadsheets. For all analysis, the second author of this paper iteratively coded the 31 interviews (using the aforementioned codebook), having regular meetings with colleagues in the team to discuss the interpretation of the codebook and reach agreement. In particular, interview coding followed the below process: three researchers coded the same interview transcript individually against the codebook. Their coding results were compared using Cohen’s Kappa coefficient with the following results: (a) agreement between Coder 1 and Coder 2: Kappa = 0.86 (very high agreement), (b) agreement between Coder 1 and Coder 3: Kappa = 0.71 (high agreement), and (c) agreement between Coder 2 and Coder 3: Kappa = 0.71 (high agreement). The overall agreement across pairs of coders was high, indicating reliability in coding (Kappa = 0.76). Areas of disagreement were mainly related to the coding of specific sections in the data. For example, the quote ‘With a help of volunteers, scientists can receive a result of laborious projects’ was coded as ‘Experience with data’, ‘How CCS works’ and ‘Sharing knowledge’ by the three coders respectively. These issues were discussed and resolved in a meeting where common understanding regarding the meaning and definition of each code was established. Also, a new code was added to the codebook regarding: ‘Displays of scientific reasoning or developing scientific inquiry’, while the ‘or NOT’ was added to the code ‘Increase Value or Gaining new value/interest’ to denote cases of decreasing value and interest in CCS.

#### Focal participants

The third author of this study led the analysis of the three focal youth’s datasets (Table 1). Analysis mainly involved an interpretative analysis of the data sets facilitated by qualitative coding, using the aforesaid codebook, the coding agreement practices, and the thematic analysis principles. Two additional researchers coded the fieldnotes, mapping key ‘action and/or interaction episodes’ of participation in CCS activities and evidence suggesting ESA learning. The coded data sets were the basis of individual memos which served as evidence-based profiles for each focal youth. Following a mix-methods logic, the memos integrated relevant results from all datasets of each youth (Bazeley, 2018), shifting the analysis from individual codes to examining relationships across participation and learning evidence.

The focal youth memos were used as a foundation of creating three individual vignettes, each one specifically addressing one of the three research questions. Anzul et al. (1997, p.70) describe that vignettes can be used to ‘highlight particular findings, or summarise a particular theme or issue in analysis and interpretation’. The vignettes are introduced in each section of the findings below (see Section 4). All vignettes provide: (a) a general description of focal youth's prior interest in science and nature; (b) briefly describe their engagement with CCS; (c) emphasise the role of the field-based activities when using or aiming to use iNaturalist; and (d) demonstrate short snapshots of learning evidence (i.e. ESA), to highlight particular findings in relation to one of the research questions.

## Findings

Findings in relation to each research question are presented below in individual sections: we provide (a) the analysis of the interview data of 31 young people; (b) evidence for each aspect of ESA development among young people, and (c) an individual vignette based on the profile of one of the three focal cases which is focused on highlighting findings relevant to a specific RQ, as a means of illuminating our findings through focal youth narratives.

### ESA manifestations as reported by young people (RQ1)

For RQ1, our findings show that overall ESA1 and ESA2 were frequently found in our data with the exception of aspects such as norms of science (ESA1), a display of scientific reasoning (ESA1), and recognition by others (ESA2) that were less often observed (see first column, Table 2). ESA3 was less frequently identified overall, with taking initiatives (ESA3) being the least observed manifestation of learning. Table 2 (see below) defines the various ESA manifestations and exemplifies these with quotes from the interview data. Components of ESA, that is ESA1, ESA2, and ESA3, often co-occurred in the practice of a science activity. In particular, ESA1 and ESA2 manifestations were often co-present in the data indicating that they are likely developing together. The following excerpt exemplifies how the development of scientific skills/using the tools (ESA1) and gaining new value about science (ESA2) co-developed when making observations in nature:

Just being more thorough with iNaturalist in terms of how useful my photos are. At first, I didn't really think to make for mushrooms, […] to make sure I document what they're growing in and make sure to get pictures of the caps and the gills. That's another thing that I've gotten better at, just being more useful with pictures I'm taking. Also, being more thorough in logging locations because it took me a while before I realized that my phone isn't always really accurate with locations and I need to be more accurate, and I need to make sure that's going right. (Learner 2 M −18)

Similarly, in the excerpt below the development of scientific skills while using iNaturalist (ESA1) resulted in a transformation of science practice shown in engaging with additional resources and people outside iNaturalist (ESA3):

**Table 2.** ESA manifestations, definitions and example quotes.

**ESA1: Environmental science content and science practice, and norms of science**		
ESA	Definition	Quote
Norms of science *n* = 8	Participants talk about norms, rules or protocols that need to be followed, while collecting or reporting data such as how to engage with organisms in respectful ways.	‘I always try to make sure I don't disturb or disrupt animals and creatures and things. The rule of iNaturalist is always to release everything once you've finished identifying and observing it. I guess other things would include trying to make sure they're really good quality pictures before they are uploaded […] When I go to find animals, a good thing to do is rather than going to look for them, you sort of wait for them to come to you. I guess walking around and rustling and making a lot of noise means that you'll disperse any possible animal, so I think waiting and really looking carefully for things is the best way to do that.’ (Learner 7 – M −18)
Scientific skills/using the tools *n* = 19	Participants describe their own engagement with any stage of scientific research, suggesting a development of basic scientific process skills for project-specific tasks, such as inferring and argumentation	‘I got a bunch of suggestion IDs for a damselfly just now. I automatically go to the suggestion thing. I basically look at different suggestions to see if some people upload the same suggestion. The most people with the same suggestion, if it's the same ID that I used and I keep that ID. If there's more of a different ID I'd change it, because more people agree on that ID. Right now, I have four people that agree that this is a damselfly, and one person that disagrees that it's a dragonfly.’ (Learner 13 – M −10)
Draws on prior knowledge and science skills *n* = 17	Participant connects prior experience and content knowledge and skills for scientific practice to the current learning environment	‘Yes, previous knowledge does help a lot like knowing how to take a sharp and good picture, because that also helps to identify, like, if you come back and look at them on the computer, you can zoom in, and it really helps identify.’ (Learner 6 – M −13)
Scientific knowledge & environmental science content *n* = 25	Participants talk about learning new content, suggesting their added or expanded subject knowledge of environmental sciences	‘I figured out how to get good at macro photography. It helped me get a lot into ants and small things like that. Basically, just the main thing about me is I just have an itch in my brain where I just want to know what everything is around me in terms of the organism and iNaturalist has let me do that quite well.’ (Learner 2 M −18)
Perception of how CCSworks *n* = 27	Participants explain how CCS works or how online platforms like iNaturalist work	‘I think the scientist collects data [in iNaturalist] since they work at the Natural History Museum. They might get to spend more time with more species and learn more.’ (Learner 3 – F −11)
Youth’s perspective on or experience with data *n* = 12	Participants understand the potential scientific value of contributions to iNaturalist or how they can be used outside the platform	‘Data just stay on iNaturalist. I know that sometimes they end up popping up on someone's blog or something as being used for research. I have had I think two or three times where people have contacted me and said, ‘Hey, I'm working on this guide, is there any chance that I could use your photo for my guide?’ I always say yes because that's really cool. Obviously, I want to get involved in that. Otherwise, I'd say after uploading them to iNaturalist, I don't really think that they usually go anywhere beyond on that at least without somebody contacting me and requesting me to go somewhere.’ (Learner 19 – M −19)
Displays of scientific reasoning *n* = 9	Participants describe or engage in scientific enquiry toward specific social purposes	‘Everybody else who uses iNaturalist, they get to see their uploaded photos. Therefore, they can comment or agree to disagree with your answer.’ (Learner 5 – M −11)
**ESA 2: Identifying own expertise within environmental science**		
**ESA**	**Definition**	**Quote**
Ownership *n* = 12	Participants suggests expertise on how to document and/or identify organisms in iNaturalist, demonstrating ownership of the scientific process to generate a record	‘During lockdown, [scientists] were saying about taking a picture of the entire plant. I like to get really zoomed up photos of specific areas and make sure that if it's, for example, a fungal infection, that the leaf is identifiable as well, or what tree it's from. I think it's really important to get a picture that shows the entire animal and maybe a zoomed-up section as well and make sure you've got it in – If it decides to land at a different angle, make sure if it looks different from above than to the side, you get a few different sets of angles on it.’ (Learner 9 – F −16)
Shares knowledge/ expertise teaching others *n* = 27	Participants take on more responsibility for communications to outside audience (peers, family, or friends), sharing knowledge of science content and skills arising from engaging with iNaturalist.	‘I talked about iNaturalist with my family quite a bit because I manage to – because I go with my nan when I walk the dogs. We get really involved trying to look for different insects and my nan's amazing at spotting insects. We've made a bug-hunting team pretty much, but yes, we've supported caterpillars. I think we've both got really interested in it and I've gone out in the garden with my mum. I think it's made us a lot more aware of what's around us and yes, it's always a really good experience. I think throughout lockdown as well, it was a very positive thing to be doing.’ (Learner 9 – F −16)
Takes on Roles or developing new role *n* = 21	Participants describe how they take on roles within the scientific activities to generate and contribute data to iNaturalist	Interviewer: ‘Is there anything you feel that you take the lead on, you are most active in?’ Young person: ‘Definitely taking the pictures.’ (Learner 3 – F −1)
Science identity Performance *n* = 27	Participants say that they got better at something related to generating and contributing data to iNaturalist	‘I can take clearer pictures […] I've learned lighting and taking clearer pictures because I have to take research-grade pictures so I've been trying to figure out how to do that.’ (Learner 16 – F −10)
Science identity Competence *n* = 28	Participants describe they feel like a scientist, or identify as someone who does science, showing perceived competence to engage in the activities that contribute to iNaturalist	‘I was in my backyard and my mum wanted me to try and do it by myself. I did that and I was trying to think about everything around me. I looked under rocks. I looked at the different plants and we put this little wood plank in our backyard so that we can flip it over so that there's species under it. That makes me feel like a scientist.’ (Learner 3 – F −1)
Science identity Recognition by others *n* = 5	Being recognised by others as a good ‘scientist’, using tools and hypothesising solutions/ideas	‘My grandma definitely knows that I am good at science. She is amazed at what I know about like all the trees and plants and everything. I taught her to be an iNaturalist user, she agreed and downloaded it.’ (Learner 28 – M −10)
Gaining new value or increase value *n* = 30	Participants talk about gaining a new perspective by valuing or taking an interest in science or nature	‘iNaturalist helps me be more attentive to what's around me, so that I can find more wildlife and it's really fun when I find something […] I learned about the jumping spider. That a female has something different from the male, it was the colour.’ (Learner 3 – F −11)
**ESA3 Developing environmental agency**		
**ESA**	**Definition**	**Quote**
Desire to become some type of scientist *n* = 10	Participants envisioning themselves to become a scientist and performing in any type of science	‘I was very hesitant about being in an in-person event with a lot of people who are more knowledgeable in science and devoted to it. Yes, I want to take it to a next step by having those in-person connections. Also, I think iNaturalist definitely pushed me to end up taking just a few environmental science or science classes during my time at college.’ (Learner 31 – M −18)
Taking initiatives to do/help/contribute to science *n* = 8	Participants describe that they started a new project, made a new observation, used iNaturalist outside a CCS programme	‘My favourite genus of snakes is Bothriechis which is palm vipers. This observation was originally marked as Bothriechis marchi which is known to be found in these mountains right here […] but I was kind of interested on why this one observation was isolated by this one valley right here from all the other observations of Bothriechis marchi.I did some investigation on my own and I read like a few papers and stuff […] so I went and got this photo in a photo editor and kind of did some changes to be able to see the scales clearly […]. I wasn't able to see the important scales […] but eventually I went, and I wrote down my reasoning for saying that this is probably guifarroi and here, the snake venom researcher from the UK said that is probably correct. That's one of the the times that I did an investigation kind of by myself.’ (Learner 2 M −18)
Describes plans to use learned scientific practice in another context *n* = 13	Participants describe an action or intention to use the tools or/and scientific practice learned for and through iNaturalist in another context	‘When I go with a picture, kind of want to know what its purposes are and what it can do. […] Because sometimes there are competitions like the – there are projects like the City Nature Challenge and I join in with them and stuff.’ (Learner 26 – M −10)
Transformation of practice as a result of agency (generative) *n* = 12	Participants describe how their daily practices have changed following participation in iNaturalist	‘I want to find out which of the US states has the greatest diversity of rattlesnakes’. I'll go state by state and look at how many species of rattlesnakes people have been able to post, I'm like 'okay, it's clearly Arizona'. For me, it's just a matter of coming up with questions and figuring out a way I can answer that. Also, other ways I can do that is: 'what's good time of the year to look for these specific birds?' I can click on the species in an area and look at the little chart that shows you how often people are posting on species and things like that. Whenever I go on a vacation or anything to a different place, I want to know what's the sort of stuff, what lives around here?’ (Learner 2 M −18)

I started iNaturalist in late 2019 and started researching the most common types of Lepidoptera and beetles in my area. Then it spread to arachnids and grasshoppers […] I tried to follow-up on my questions and investigate the animals […] I googled information and bought books on insects. I also have friends who are [biologists] and they taught me a lot of things. (Learner 30 – F −19).

In terms of all three components of ESA, the following young person explains how they developed knowledge and expertise in identifying pollinators (ESA1: Scientific knowledge; ESA2: Science identity performance) and how this led them to study species variations and develop an interest in entomology (ESA3: Taking initiatives):

I've been particularly interested in my butterflies and bumblebees. I've been tracking the different types of bees we've been seeing throughout the year, from March until now. For example, I think it was only a couple of weeks ago, we started getting Common carder bees coming back again. Just studying variations in them. It's got me a lot more into that entomology side of it especially. (Learner 9 – F −16)

#### Vignette 1: ESA learning and the blended learning environment of iNaturalist

Vignette 1 illustrates, through the story of a young person, Daniel who is 12 years old, specific CCS learning outcomes with ESA1, ESA2, and ESA3 often co-occurring in the practice of a science activity.

**Daniel, 12 years old**

Daniel liked ‘walking a lot, looking at animals, catching lizards’ and enjoyed reading news articles online (Source: online and field-oriented interviews). He was interested in herpetology, and through [programme X] (a community and citizen science programme at his local Natural History Museum), Daniel became familiar with iNaturalist, used his previous experience exploring wildlife, and expanded his expertise on the subject (field-oriented survey and interview). According to Daniel, iNaturalist was ‘the only platform I use for documenting nature’ and had been using it for a couple of years (Source: online-oriented survey). iNaturalist gave purpose to his fieldwork activities to record species occurrence in local areas, gave him access to taxonomic facts of species, and a community of people with similar interests to his. Daniel described, ‘I’ve learned a lot more about identifying the things I find. I’ve gotten a lot of tips and tricks through iNaturalist about animals in general’ (suggestes ESA1) (Source: online-oriented interview), a finding also described and observed during field surveys near home (field notes 1). iNaturalist was a mediator between his field expertise to find salamanders and other small creatures and his drive to identify species, as observed multiple times during the field surveys.

He was aware of the scientific value of his contributions to iNaturalist, he said ‘It's contributing by finding little things, all the little creatures’ (Source: field-oriented interview); he would usually ‘check all the comments on [his observations in iNaturalist] to make sure I didn’t identify anything wrong’ (Source: online-oriented interviews) (ESA1). Daniel did not feel confident to verify observations from other iNaturalist users (Source: online-oriented interviews and field notes 2). This was contrasting with the confidence he demonstrated during field surveys and while talking to our field researcher about his strategies to find and attract salamanders, which became more sophisticated over time (ESA2) (Source: field notes 1–4). Daniel surveyed his local neighbourhood with family members, which gave him an opportunity to take on roles within fieldwork and put his expertise in practice. He saw himself as a ‘a combination of finder and picture taker’ (ESA2) (Source: field-oriented interviews). He also drew confidence from iNaturalist, he said, ‘every time I have a correct identification that’s Research [Grade] it just proves to me I do know stuff. I’m really happy when I have a correct identification. It’s a nice little confidence boost’ (Source: online-oriented interview).

During his participation in the CCS programme, he felt like he was ‘doing science’, and he explained that ‘posting my observations and contributing to the effort to learn about biodiversity in my area feels like science, as it gives me a chance to interact with scientists and help with their work’. (ESA2) (Source: online-oriented interview). Daniel was perceived as inspired by meeting and doing a field survey with one of the museum scientists who specialised in Herpetology (field-oriented interview). Daniel added that he felt like a scientist because ‘Just by contributing to the work effort of cataloguing all the things in Los Angeles, I felt more connected to the process. It felt less like a hobby and more like something that was actually helping the world.’ (ESA2) (Source: field-oriented interview).

Through CCS, Daniel laid a foundation for more than individual change (ESA3), he said ‘I’d say that we’re responsible for most if not all of the [iNaturalist] posts in our direct area’ (Source: online-oriented interview). Further, Daniel planned to continue participation in theprogramme, and used iNaturalist in his everyday life when walking his dog (ESA3). He had also joined a BioBlitz where he used iNaturalist, and species identification debates, such as one on slender salamanders, on iNaturalist (ESA3) (online and field-oriented interviews).

### Types of participation and development of ESA manifestations (RQ2)

We identified five different ways of scientific participation on iNaturalist: (1) *Exploring and Discovering*, (2) *Observing*, (3) *Documenting and Recording*, (4) *Identifying species*, and (5) *Communicating*. These types of participation were observed in (a) online and (b) field-based settings: (a) online on the iNaturalist platform enabled by design affordances such as commenting and identifying observations of others and scaffolding the process of identification using machine learning algorithms, and (b) offline in the field while young people engaged with nature. Table 3 defines each type of participation, noting whether it has been manifested in the field and/or online settings and presents an exemplary quote showing how it maps with specific ESA components. For example, *Online observation* refers to watching/looking at photos of species online using integrated tools and features on the iNaturalist platform (e.g. exploring the observations list using the ‘Map’ tab) while *Field-based observation* refers to using senses (e.g. touching, listening) to find, notice, watch wildlife, with or without the use of tools (e.g. magnifying glasses), and with or without guidance from others.

**Table 3.** Types of participation and ESA manifestations on iNaturalist across field-based and online settings.

**1. Exploring and discovering (*n* = 26)**		
Definition of participation	Related ESA	Example quote
**Field-based exploring:** involves discovering new things such as new species. Exploring a habitat, searching for wildlife, e.g. looking under logs, turning over rocks, collecting a specimen (e.g. catching a bug or collecting seaweed); this can be with tools (for example nets) or without them.	ESA1 – Scientific knowledge & Environmental Science Content	‘I went out of London and up into the countryside. We went to a Royal Society for the Protection of Birds nature reserve. We've just recently purchased a pair of binoculars and we've been watching this woodpecker, and she's made a massive hole. We took the binoculars and we saw loads of stuff like oystercatchers.’ (Learner 11 – M −12)
**Online exploring (iNaturalist and others)**: Exploring or searching for information (reading, viewing images etc) (a) randomly out of curiosity, (b) intentionally i.e. with a purpose in mind such as to answer a specific question, (c) update or expand present information. Moreover, young learners, employed other online tools such as Google search as an online discovery method.	ESA1 – Scientific skills/using the tools	‘I tend to turn to a lot of scientific websites and pages that list animals and they give explanations and things. If I want to learn, I basically research and write down discoveries and things. If I want to learn more about the species I look it up using the internet, which is a very useful tool. I do have lots of books of animals and wildlife[..] While I do read a lot of books, I do tend to use the internet for – If I want to find one specific species, I tend to use the internet as my primary source of research for my findings.’ (Learner 7 – M −18)
**2. Observing (*n* = 3)**		
**Field-based observation:** using one's senses (e.g. touching, listening) to find, notice, watch wildlife, with or without the use of tools (e.g. magnifying glasses), and with or without guidance from others.	ESA2 Gaining new values/interests	‘Trees are around, figure what are the most predominant species of trees definitely get that. After that, I see whatever insects happen to be around flip over any rocks or logs. Keep my eye on the trees to see what kind of birds were flying around. A lot of interesting stuff and there were so many butterflies and [crosstalk] a parasitic wasp.’ (Learner 2 M −18)
**Online observation (iNaturalist):** similar to the field-based observation, but the sense one can use online is watching/looking at photos of species using integrated tool(s) and features on the iNaturalist platform (e.g. exploring the observations list using the ‘Map’ tab)	ESA2 – Sharing knowledge ESA2 – Takes on roles	‘iNaturalist is helpful when people supported my observation and then also say I support this, provide a reason. Then, I also noticed the bird's legs are a certain color.’ (Learner 21 – F −10)
**3. Documenting and Recording (*n* = 18)**		
**Field-based documenting and recording** of organism/species by generating evidence such as a photograph or writing on recording sheet (no reference to submission to a dataset or sharing with an expert for submission to a dataset)	ESA2 – Competence	‘I'm on a hike and I have to take a photo of something that's maybe out of reach, I'm like, ‘This is for research.’ During my internship, definitely because I had a clipboard, a GPS and a pencil, and wrote everything down. Yes, it definitely makes me feel good when I make some observations. I feel like a scientist.’ (Learner 12 – F −18)
**Online documenting and recording (iNaturalist):** uploading pictures of organisms to iNaturalist adding relevant information such as place, time etc.	ESA1 – Scientific skills/using the tools	‘Every two days, we do observations using iNaturalist […] search for the observation once you took the picture and then you press it. You just press ‘observe’ and then there's something on this side. It's like a picture of mountains and then you press it, you go to photo stream and then press the observation of the bug that you took a picture of. Usually, it's all the way at the bottom of the stream. You just press it and do all the other stuff.’ (Learner 27 – M −9)
**4. Identifying (*n* = 25)**		
**Field-based identifying:** establishing or indicating what something is such as species name, common name. Example: identifying horseflies (based on previous knowledge/ indicators). Identifying organisms is by adding a name to it (e.g. taxon or species) with the help of more knowledgeable others	ESA2 – Sharing knowledge ESA2 Gaining new values/interests ESA2 – developing new role	‘I got one comment from someone who I know in town actually, who is a botanist and knows a lot about trees and plants and all animals, really everything. I had a picture of a birch tree down in the park and he explained that – I thought it was just a white birch or a paper birch or something, but he said that all of the birch trees at the waterfall park are actually a mix. They've been cross-bred and they're a different kind. I thought that was really interesting to learn about. I don't know where that would be in all of my observations, but – Oh, you can search it. Where can you search? Oh, here? I can try and search for it.’ (Learner 25 – F −15)
**Online identifying (iNaturalist):** selecting species name, with support from a social and technology-assisted scaffolding process of identification embedded in iNaturalist.	ESA1 – Scientific skills/using the tools ESA2 Gaining new values/interests ESA2 – Sharing knowledge	‘iNaturalist chose is really helpful with things that you don't know and the photo recognition. Either it's visually similar or it's seen nearby or it's both of those things and they tell you whether or not and give you suggestions as to what it could possibly be based on the photo itself, which is really cool. Normally, they'll give you maybe five, and I look at the top three, and I look at other people's observations. I click on them and I see does it doesn't look like what this person saw, and then I'll go to the next one and I'll do that again. It's really helpful to not see the really nice, crisp photos of them, the Google search images one. It's helpful seeing other people's observations.’ (Learner 12 – F −18)
**5. Communicating (*n* = 25)**		
**Field-based communication:** instances in which youth shared science related content, results or findings of their work at a CS programme as part of the activities (i.e. wrap up session) or to outsider agents who are not part of the programme. Individuals such as classmates and teachers are examples whom young learners mentioned they have encountered and communicated with and they helped them in learning about CS	ESA2 – Sharing knowledge ESA2 – Competence	‘I explained to friends a lot of things about butterflies, it is what I learned the most using iNaturalist. Also, because they are the most “socially accepted” insects.’ (Learner 30 – F −19)
**Online communication (iNaturalist):** Providing or receiving information within iNaturalist: (i) Own observations: comment on one's own image and/or respond to comments left by others on this image, (ii) Other people's observations: comment on other users’ observations, or outside iNaturalist from scientists in organised events such as BioBlitzes.	ESA2 Gaining new values/interests	‘I tried to get every angle imaginable of horsefly, no idea how to identify this fly. This person, Arturo, he is really good at identifying horseflies. I sent it to him and I said, “Can you help me out with this?” I uploaded all these angles and he was able to tell me what species it was.’ (Learner 19 – M −19)

**Figure 2.**
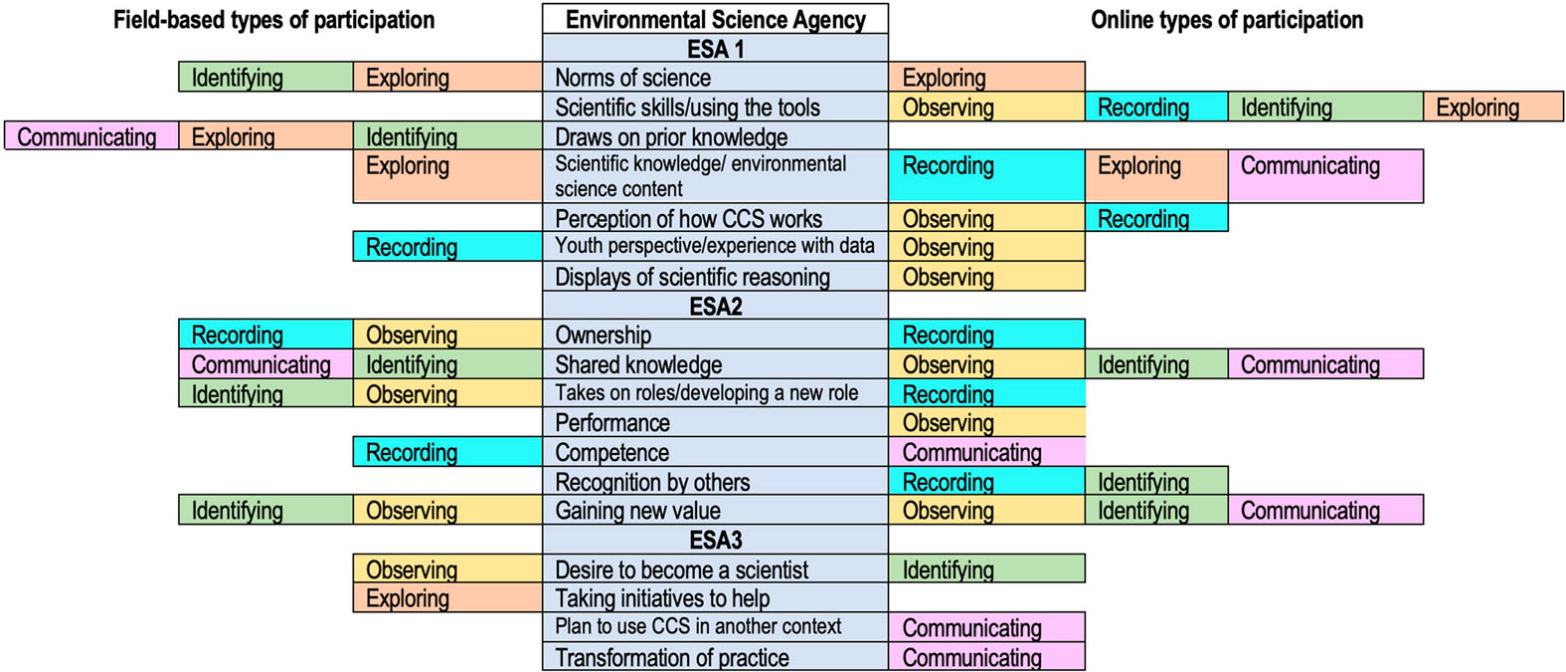
Mapping of types of hybrid participation and learning outcomes as presented in ESA.

Table 3 is complemented by Figure 2 mapping types of participation with specific forms of ESA. The analysis resulted from querying interview data, after being coded, as to which ESA manifestations are observed within each type of participation. This analysis resulted in a matrix that mapped types of participation to specific aspects of ESA, suggesting that specific participation patterns in science activities result in specific learning outcomes, as reported by participants.

In particular, the development of ESA3 is facilitated by a combination of field-based and online participation types in scientific practice, including Identification and Communication via iNaturalist as well as Exploring and Observing species in the field. For example, the desire to become a scientist, described by some youth, co-occurred with making observations in the field and identifying species on iNaturalist. In terms of ESA2, this is found to be facilitated by nearly all of the proposed types of participation, with the exception of Exploring in the field. It is not clear why exploring in the field being a practice-based and hands-on experience was not shown to support learning. It may be the case that additional data are needed to evidence this relationship, or this may be explained by no reflective elements being present while exploring. Hands-on activities (referring to students using material and engaging in practical work) may not be enough to support learning, especially if ‘minds are not on’, that is learners do not have opportunities to reflect on their practice and make sense of it. Hands-on activities should be accompanied by opportunities to raise questions and explain phenomena under examination (Furtak & Penuel, 2019) rather than being seen as an end themselves. Such processes should acknowledge and build on learners existing, yet evolving, conceptions of reality, allowing them to produce their own knowledge (Southerland & Settlage, 2019).

With regards to the development of a science identity shown in increased competence and performance (ESA2), field-based recording such as taking a picture of a species and communicating with others about it on iNaturalist were shown to enable development of science competence whereas observing photos of species on iNaturalist enabled development of science performance. In regards to ESA1, exploring in the field was particularly prominent; it was shown to support the development of scientific knowledge and understanding of the norms of science and enable activation of prior knowledge. In relation to ESA1, the only type of participation that was not found to enable learning outcomes was observing species in the field; this may either suggest that noticing species with no additional information e.g. what the species is, might not encourage learning compared to combining observing with identifying, or it may be the case data have not been collected in this study to confirm this relationship.

In some cases, certain types of participation inhibited further learning or ESA development. In particular, a young person explains how exploring or searching for information and communicating with other iNaturalist users via the iNaturalist mobile application was not straightforward:

With the mobile app that I use, I find it very difficult to search for individual people, people accounts, and observers, to try and find them and see what they've observed. I usually feel it's quite difficult for me to interact with specific people that I want to find. (Learner 18 – M −19)

A second participant explains how having others identify their observations would engage them further with iNaturalist activities: ‘There's some of my observations that it makes me sad when I take a really cool picture of something, but no one looks at it anymore’ (Learner 12 – F −18). Another participant commented on sharing their account with their family and how this may delay receiving information from iNaturalist about identifications on their images: ‘If it's my own account it will make it easier for me to check these identifications. Because it's on my phone. If it's with my mom's phone, I won't find out until she tells me’ (Learner 26 – M −10).

An emerging finding from the interview analysis was the significance of external tools not related to iNaturalist that scaffolded young people's learning and participation. In particular, we observed youth leveraging offline and online resources, other than iNaturalist, to help them participate and learn from iNaturalist. Young people search for information in online websites such as the eBird platform: ‘Like if I see a Sandpiper that I don't know what it is, I would put it on iNaturalist […] I also use eBird […]. It's got all the birds of Eastern Canada’. (Learner 6 – M −13) (ESA2: development of new roles). Other times they would use online search engines to support the process of species identification. As explained: ‘If I want to find one specific species, I tend to use the internet as my primary source of research for my findings’ (Learner 7 – M −18) (ESA1: Scientific skills using the tools). Another participant explained their seeking of information outside iNaturalist by not receiving a specific answer on iNaturalist:

Often when I post to iNat, it doesn't give me an exact answer. So, I like to research it more so I can try and figure out what it is. […] I look at responses and I google. I like to try and find reliable websites. I've been taught to do that at school. (Learner 17 – M −13)

Another participant explains how communication with family members or friends helped with developing their knowledge about citizen science: ‘My mom, my dad, and my grandma, I taught them how to use iNat, and what to do with it. My grandpa used to teach me a lot of words about science, so probably from there I know about citizen science’ (Learner 28 – M −10). Andrea’s story in Vignette 2 illustrates how specific participation types enabled certain forms of ESA.

#### Vignette 2: Developing ESA by engaging in the scientific types of participation

Vignette 2 presents the story of a young participant, Andrea who was 10 years old, and how she developed ESA by taking part in CCS activities.

**Andrea, 10 years old**

Andrea was interested in science, geology books, and nature walks; she also said: *I’m good at using technology, and I'm good at asking questions*, which she believed was crucial to do science (field-oriented interview). Her family had introduced her to iNaturalist when she was six years old, but back then she could not work out how to use it (online-oriented interview). About four years later, Andrea had become an eager user of iNaturalist and was further involved in CCS activities and events organised by her local NHM. Andrea was Daniel’s sister and participated in [programme X] together, during which she demonstrated learning outcomes for each of the ESA components.

Andrea developed ESA as she engaged in the scientific types of participation encouraged by the species occurrence focus of [programme X] and iNaturalist. She described

I like bugs because you don't see them if you're just looking across the path, but once you get in close, there are a whole lot of bugs. There's way more life on a bush that you don't see unless you're specifically looking. (field-oriented interview)

On multiple occasions, Exploring and Finding and Observing her surroundings and seeking to Identify and Record the species she found led Andrea to think scientifically by posing new questions or gain understanding of species habitats (ESA1) (field-oriented interview; field notes 1, 2, and 4). Andrea sought to answer her questions by Communicating with knowledgeable others; she described:

I would investigate [my questions] usually by asking people or reading books. A lot of the time when I was at a [programme X] event, I would just ask a scientist … . I would also upload it [on iNaturalist], and I would read books about it, that kind of thing. (field-oriented interview)

Communicating her findings and questions to others gave her opportunities to gain and share knowledge about science, boosting her confidence and competence to participate in science (ESA2) (online-oriented post survey; field notes 1, 2). Communicating with NHM scientists also gave her access to new networks and CCS projects, after one of the [programme X] meetups, Andrea and her family joined a different project focused on recording bats living in local backyards (ESA3) (field-oriented interview).

Of course, Andrea’s engagement in the scientific types of participation were linked to other learning aspects such as gaining content knowledge, developing skills for scientific practices (ESA 1), and reinforcing her confidence to teach others and becoming better at participating and contributing to the recording of species occurrence (ESA 2). Among these, a distinctive learning outcome was that Andrea developed environmental agency (ESA 3) as a result of Exploring and Finding for wildlife in local areas.

Besides taking part in [programme X], Andrea joined other projects on iNaturalist (ESA 3). She said: ‘I did the NHM projects and then I also found some other [projects on iNaturalist] for the things that I was finding a lot of’ (field-oriented interview; field-oriented post-survey). Andrea was repeatedly finding mushrooms, fungus, and slime and wanted to Record them and Identify them (online and field-based oriented interviews). She enacted her agency to join an iNaturalist project on mushrooms, she described:

I know more about [mushrooms] than I did before. I'm looking into some of them that I'm very interested in. I think iNaturalist has helped me because it gives me a starting point … you can't just google what a photograph is or something, but iNaturalist gives me a name and sometimes also an article. (field-oriented interview)

Through Exploring and Finding and Observing wildlife in her local areas and using iNaturalist to Identify and Record the species she found, Andrea was encouraged to develop her interest in species occurrence, learn about the species she found, pursue questions scientifically, and widen her view of the biodiversity where she lives.

### Previous science experiences and ESA manifestations (RQ3)

A number of interview and survey questions asked participants about their prior experience/interest in science including whether they are using tools and technology to access science, any experiences with science or natural science such as working with a club, museum or institute and any other personal interest/hobbies. A majority of participants (*n* = 17/31) mentioned an a priori interest in science, citizen science or nature, while some participants mentioned working in science centres and museums. These prior science experiences explained or motivated participation in iNaturalist as explained below:

I first started on the computer because I did an internship at Pepperwood Preserve in Santa Rosa. The whole internship was based on iNaturalist. We would go out with digital cameras and upload it on our computers later. I really started with the website itself but found out there was an app. Now that I'm not a part of that internship, it's really easy to use. (Learner 12 – F −18)

Another participant explains how participation in another CCS platform (eBird) informed them about iNaturalist:

I did actually come from eBird that I found iNaturalist. I had a big group server, group chat for people that used eBird and somebody had brought up iNaturalist and introduced it to the chat as a way to learn about wildlife aside from just birds. They ended up making a separate chat for iNaturalist so I joined pretty early on. I think I was actually the third person to join. We used it pretty extensively and it started to take up a lot more of my naturalist time, I guess. I got more into it through that. eBird.org. It's run by Cornell. It is very helpful for bird information. Also, there's hotspots listed where you can see what people are seeing there which is fairly helpful. (Learner 19 – M −19)

In addition, as shown in Table 4 these prior science experiences scaffolded learning, in particular specific ESA manifestations related to ESA1 and ESA2. For example, regarding ESA1, they enhanced science learning and understanding about the environment (ESA1: Scientific knowledge & Environmental Science Content):

I subscribe to a lot of different things that are science related. Also, I follow a lot of other science fields outside of biology that I'm into. iNaturalist let me get a bit better at taxonomy, just learning about taxonomy, being able to look through it, I find more things to learn about that I find interesting, that sort of thing. (Learner 1 F −13)

and regarding ESA2, they contributed to better science performance and development of science identity:

[iNaturalist] just let me do more of what I like to do. The way I learn science often isn't exactly the best one for school. For me, I'll just find a topic and do a lot of research into it for a while and once I feel like I have a sort of a cursory understanding I go: “Yes, it's cool.” Now I feel like I know a lot about this. (Learner 2 M −18)

Vignette 3 presents the moments in which a young person used their prior knowledge to support their field surveys and identification of species.

**Table 4.** Relationships between ESAs and prior science experiences.

Prior science experiences	ESA manifestation	Examples quote
Interest in nature	ESA1: scientific skills/using the tools	‘On Google Play Store, there’s an app that identifies leaves and stuff like that. So, I understand how it works, and I know that on the project. I'm zooming out, and there's a lot of observations near us.’ (Learner 23 – M −7)
Interest in nature	ESA1: Scientific knowledge & Environmental Science Content	‘There are lots of bugs coming to our house because we usually grow lots, we do like farming and different things. Therefore, I take pictures of insects mainly because all the world is 19% bugs, and so iNaturalist sort of focused on the bugs.’ (Learner 26 – M −10)
Interest in science	ESA1: Scientific knowledge & Environmental Science Content	‘I subscribe to a lot of different things that are science related. Also, I follow a lot of other science fields outside of biology that I'm into. iNaturalist let me get a bit better at taxonomy, just learning about taxonomy, being able to look through it I find more things to learn about that I find interesting, that sort of thing.’ (Learner 1 F −13)
Interest in Citizen Science	ESA1: Perception of how Citizen Science works	‘I was first introduced to how Citizen Science works when with this habitat restoration group that employ citizen science by having local volunteers help them with creek water quality monitoring. They advertised that as a citizen science opportunity. It's an easy way for everyone to participate in science, no requirements but a person can observe things that happen in the world and record that data and provide that data to others, as we do on iNaturalist.’ (Learner 22 – F −20)
Work at a museum	ESA2: Shares knowledge/expertise teaching others	‘While I was working in the [museum X], I was in this programme where they'd take teenagers and we'd act as docents […] When I downloaded the iNaturalist app […] I just went through all of my personal archives, the photos that I had taken over the past six years and I uploaded as many of them as possible. When I am curious, I’ll go to people I know who are good at identifying anthropoids or whatever insect. I’ll tag them or tag a couple of people if it’s an ant I’ll definitely tag Doctor [‘ What is this?’ If it is a bird, I’ll reach out to someone online and ask,’ Okay, what’s this?’ Generally, just a matter of taking the pictures and then reaching out to people I know.’ (Learner 2 M −18)
Interest in species identification	ESA2: Takes on Roles or developing new role	‘I did use eBird to see what birds people have seen in my area [..] and I use allaboutbirds.org to identify birds because they have a database of different bird species. For each bird species, they talk about how to identify it, maps of its habitat, that sort of thing. I use it more for research. I have used iNaturalist because it is easier for me to use than eBird. Some plants just happened to grow in the city so that's why I started using iNaturalist again to contribute data to what is around.’ (Learner 22 – F −20)
Interest in science	ESA2: Science identity Performance (Gaining expertise and develop skills in using the tools)	‘[iNaturalist] just let me do more of what I like to do. The way I learn science often isn't exactly the best one for school. For me, I'll just find a topic and do a lot of research into it for a while and once I feel like I have a sort of a cursory understanding I go,’ Yes, it's cool.’ Now I feel like I know a lot about this.’ (Learner 2 M −18)
Interest in science	ESA2: Science identity Recognition by others	‘Well, my grandma definitely thinks that I am good at science. She is amazed at what I know about like all the trees and plants and everything […] She doesn't even believe me when I say what some of them are until she scans it herself – I taught her to be an iNaturalist user.’ (Learner 28 M −10)
Interest in science	ESA2: Science identity Competence (Self efficacy and confidence)	‘I learned about cell signalling and cell division in bio classes that's not exactly the biology that I was super interested in. I'll be looking into our behaviour and how that works and taxonomy, they learn how various things are organised but it's not as in-depth as what I need for my bio classes. On iNaturalist, I am more confident just talking to people, talking to scientists like on twitter. Before this I wouldn't reach out to people where everybody has questions to ask.’ (Learner 2 M −18)

#### Vignette 3: using prior knowledge to support field surveys and observation in iNaturalist

Vignette 3 presents the story of Carol, 11 years old and how her prior experiences supported field surveys and observation tasks on iNaturalist.

**Carol, 11 years old**

Carol liked science and was enthusiastic about nature. She described having good grades for science in school, she liked learning science outside of school, and wanted to know about plants and species that were new to her (field and online-oriented interviews). Carol reinforced these interests when she joined [programme X] and, demonstrated ESA1, ESA2, and ESA3 learning while taking part in [programme X]. Among these findings, the evidence shows that Carol posed questions and explanations that suggested scientific reasoning (field notes) and she used prior knowledge to understand her observations of and identify species, both important aspects of ESA1 that suggest ESA learning can also be stimulated and developed through prior experiences and knowledge.

Carol believed her science learning from school had helped her be more aware of the species she could find in a certain climate or area (online-oriented interview). Our field researcher who accompanied Carol in four of her surveys documented nine instances in which Carol used prior knowledge in specific ways to support her field-based and online participation (ESA1). For example, Carol guessed the presence of slugs by noticing ‘slime trails’, using her prior knowledge on slugs to find and create evidence for iNaturalist (field notes).

Carol showed knowledge of insects and their interdependence with plants. Most examples of Carol using her prior knowledge involve an insect and a plant or a topic around plants; in fact, Carol’s observations in iNaturalist show she predominantly recorded plants (log files). Carol was observed identifying aphids with confidence and explaining she had seen them when cutting roses at a Garden Club she used to join prior to [programme X] (field notes). She also knew that tropical milkweed attracts monarch butterflies (field notes) and that insects can look like a leaf to camouflage from predators (field notes).

After having taken part in [programme X] for several months, Carol was observed using her scientific reasoning skills and prior knowledge to solve things that puzzled her such as why she had seen multiple caterpillars and then butterflies in the backyard, but it puzzled her that she never found chrysalides in plants (ESA1) (field notes). The evidence shows that her long-term participation in [programme X] gave her multiple opportunities to use her prior knowledge in field surveys. Moreover, Carol’s mum and our field researcher encouraged Carol to pursue her questions and supported her understanding of other evidence (slime trails or chrysalis), which in fact count as evidence for an observation in iNaturalist.

## Discussion

### Addressing research questions

In this paper, we evidenced through 31 in-depth interviews, three of which were complemented by observations in the field and prior to field/post-field surveys, how participation in CCS online and in the field, on the iNaturalist platform, can promote environmental science learning, as described by the ESA framework, of young people aged 7–20 years old (RQ1). We also illuminated how the design of hybrid CCS programmes can enable, and in some cases, hinder participation and blended learning in CCS (RQ2) of young people including those with limited prior science learning experiences (RQ3). These findings are discussed in the following paragraphs.

In regards to RQ1, young people who participated in CCS using the iNaturalist platform reported learning benefits from participation mainly related to the development of scientific thinking skills and knowledge in particular how CCS works, their experiences with using data, their skills of using tools and their knowledge about the environment as well as the development of roles in the practice of science including gaining a new value, taking up a new role, enhanced science competence and performance and sharing of knowledge with others. For some young people, engagement with activities on iNaturalist led to the development of environmental science agency evidenced in plans to use science in another context, desire to become a scientist, and initiatives to help others and transform their own practice. It could be argued that the online dimension of iNaturalist, field-based activities, as well as the blend between the two are learning contexts that enable and foster youth science learning. These findings align well with existing studies reporting learning outcomes of youth's participation in CCS both online and in the field (Herodotou et al., 2022; Ballard et al., 2017; Harris & Ballard, 2021). A study focused on young people’s participation in environmental and field-based CCS identified that access to tools for fieldwork, guidance to use these scientifically, and repeated use led participants to try different roles and gain science disciplinary skills (Ghadiri Khanaposhtani et al., 2022). Moreover, a study on young people’s learning across field-based CCS programmes and an online platform (Zooniverse) found that field-based scientific practices (such as those encouraged by iNaturalist) enabled participation and learning through role-taking in CCS, and this was a unique learning outcome in contrast to the learning evidence of the online CCS participants (Benavides Lahnstein et al., Under review). Overall, engagement with authentic biodiversity research as conducted on iNaturalist, helped young people to become more ‘competent and knowledgeable actors’ (Ruiz-Mallén et al., 2016); the informal and hybrid implementation of CCS enabled forms of learning that are not yet supported in CCS implementations in formal education (Williams et al., 2021), including the development of youth's science identity and agency.

In regards to RQ2, science learning outcomes were enabled, yet in some cases inhibited, by specific types of participation, the latter supported by design features of iNaturalist and external resources such as more knowledgeable others and other learning tools. As shown in Table 3, half of the proposed types of participation took place online and the other half in the field, suggesting that both settings separately enable learning. The ‘blend’ was shown to extend field-based observations; it was mainly evident in participation types were field-based activities were a prerequisite for online participation such as searching for information regarding a species observed in the field, recording and identifying it and communicating about it with others online. Proposed participation types were informed by Lorke et al. (2021) work documenting participation in field-based CCS events (BioBlitzes). Our analysis showcased that the following forms of participation *Exploring*, *Observing, Identifying, Documenting and Recording,* originally observed in field-based settings (See Lorke et al., 2021) have been also documented in our study in the hybrid environment of iNaturalist. Yet, it is noted that online participation types are documented for the first time in this paper. Compared to Lorke et al. (2021) work, we named a new form of participation *Communicating*. In Lorke et al. (2021) communication instances formed part of the process of *Identifying* species as often more knowledgeable others helped young people to identify species. In this paper, *Communicating* does not only refer to support from more knowledgeable others but also includes an element of ‘teaching others’ by sharing science-related knowledge with schoolmates, family, etc, and also young people helping others to identify species by commenting on their observations shared on iNaturalist.

The mapping of types of participation with specific learning outcomes (Figure 2) elaborates on and extends the working framework first proposed and presented in Herodotou et al. (2022). The latter followed the same processes of data collection (interviews) and analysis (code book, mapping of participation types to ESA manifestations) as the present study, yet participants in the former were young volunteers from the Zooniverse platform. iNaturalist insights from this paper extend the applicability of the original framework beyond a single crowdsourcing platform (Zooniverse) to CCS platforms that target biodiversity monitoring. Compared to the types of youth participation identified in the Zooniverse platform (Herodotou et al., 2022), iNaturalist presents some unique and overlapping features. In particular, *Observing* that is, using senses to notice and observe nature, is found to be unique to iNaturalist. This could be explained by the focus of iNaturalist on capturing and identifying biodiversity, for which a close observation of nature is needed to find, document and name species. The overlapping types of participation between the two CCS platforms are *Exploring and Discovering* and *Communicating*; young people search for information both within and outside the two CCS platforms and also use communication features and more knowledgeable others to discuss and complete tasks. The participation types of *Documenting and reporting* and *Identifying* align with *task-based* participation on Zooniverse. These types of participation describe the tasks one is expected to pursue when using iNaturalist. These tasks are rather distinct to the tasks pursued on Zooniverse; tasks on iNaturalist are focused on data collection and identification whereas on Zooniverse are focused on data analysis.

Scaffolding has been a significant dimension of participation and learning; this was enabled by the iNaturalist design affordances including the AI-based species recommendations and community identification as well as interactions with more knowledgeable others both online and in the field. It is noted that some of the participants of this study were recruited in field-based CCS museum-led events that provided basic facilitation such as general short training sessions and guidance on how to use iNaturalist. Following these events, participants could continue using iNaturalist on their own or take part in long-term CCS events where they could have further opportunities for facilitation. Facilitation, in particular when scientists explained clearly and repeatedly the goals of a programme during an event was shown to result in more engaged youth participation, while facilitation in using the tools enabled participation in different aspects of CCS (Ghadiri Khanaposhtani et al., 2022).

Similarly, the use of a mobile app while detecting and monitoring species in nature had a positive impact on the outdoor experience and associated learning implications. The iNaturalist app enabled youth to enhance or augment their outdoor experience by accessing information about observed species and the broader ecosystem they belong to. Should observations be shared with others while in the field, this could further enhance learning through information received by other iNaturalist users. As shown in this study, a mobile app was a valuable tool that supported learning about the natural world. These observations align with existing studies showing that the use of mobile technologies increase motivation, engagement and fun, interest for learning about a specific location, and knowledge about a place, encourage further exploration of outdoors (which can lead to greater awareness and care of nature), and enhance practical skills such as problem-solving and navigation skills (van Kraalingen, 2021). While mobile technologies can help with interactions with the surrounding environments, extra support may be needed to make connections between app concepts and the physical world (Land & Zimmerman, 2015). In this study, we observed some youth utilising external tools to complete a task on iNaturalist such as searching for information online, accessing other biodiversity websites and asking family members for help. These findings suggest that the design of biodiversity monitoring apps such as iNaturalist could be further enhanced to better support young people's interactions, resulting in enhanced learning outcomes.

In terms of young people who currently take part in hybrid CCS and are likely to enjoy learning benefits (RQ3), our analysis showed that slightly more than half of participating youth reported a prior interest in science, CCS and nature or were working in science centres and museums. These experiences and interest enabled participation in iNaturalist and helped complete tasks from which learning was reported. A follow-up exploratory analysis (not reported in this paper) showed that these prior experiences may relate to specific types of participation. For example, experiences of working in a science setting coincided with recording tasks, suggesting that work experiences may encourage or scaffold recording practices. Overall, all prior experiences reported in this study were shown to relate specifically to observing and exploring, suggesting that science curiosity and interest are likely common characteristics across prior experiences and CCS participation. Of special interest is the fact that nearly half of the participants did not declare any relevant prior science experiences and interest, yet they reported science learning from participation in hybrid CCS, emphasising the significant role CCS can play in engaging youth with rather limited science experiences with biodiversity learning. This contradicts with studies examining youth participation on Zooniverse (Herodotou et al., 2021b; Herodotou et al, 2022) and reporting that most young people had significant prior science experiences that supported their participation and learning. In the case of iNaturalist, it could be argued that young people with limited science capital, such as scientific literacy, science-related values and knowledge, connections with science activities out of school (Archer et al., 2015) could engage and complete CCS tasks. iNaturalist could be seen as ‘opening the door’ to science to those youth who have no other science experiences. Compared to other forms of CCS participation, the hybrid nature of iNaturalist can spark curiosity and give young people meaningful opportunities to enact agency.

### A blended learning framework for biodiversity monitoring in CCS

In this paper, we introduce a blended learning framework about biodiversity monitoring in CCS which has been derived from studying self-regulated forms of learning, that is, how and when young people choose to engage with nature and their surroundings, engage with tasks and seek help, if needed. The framework consists of three descriptive characteristics - these are the features of the learning experience - including: (a) *Settings:* online (website, app), field-based (outdoors, nature and surroundings), the ‘blend’, (b) *Tools:* a mobile app and website enabling species monitoring; external tools including other online websites, online search for information, knowledgeable others (family members, etc), and (c) *Scaffolding:* community support to species identification; AI support to species identification; communication with scientists in field-based events and via an online biodiversity platform. In addition, it presents the activities learners are asked to take part in and complete, manifested in participation types: authentic data collection and analysis including exploration and observation, documentation and identification, and communication activities. The components of each activity are specific tools, settings and scaffolding as described above. Activities result in the following science learning outcomes: (a) development of environmental science agency enabled by identification tasks, communication with others online, exploring and observing species in the field; (b) development of science identity (performance and competence) enabled by engagement with nature to observe, document, record and identify, as well as communication with others; (c) gaining knowledge about science enabled by all above activity types.

The above components could guide the design of hybrid or blended CCS biodiversity programmes, especially those aiming to engage young people with science. Aspects of the framework could also support environmental-related activities in informal and formal settings (e.g. schools), specifically the use of biodiversity monitoring apps to enhance and augment the learning experience in the field by setting as learning objective the observation, recording, and identification of species outdoors.

Considering findings from other studies analysing young people's log files from iNaturalist (Aristeidou et al., 2021a), young people mainly make observations of specific species such as plants, birds, molluscs, and arachnids with very few identifying species or communicating with others. This suggests that we should identify ways for young people to engage more with identification and communication tasks, to enable development of environmental science agency. The mapping of types of participation to learning outcomes (Figure 2) could inform the design of CCS programmes by offering a fine-grained representation of how a CCS programme should be designed to achieve specific learning objectives, including participation types less likely observed such as identification of species shared by others.

### Limitations and future directions

Participation and learning outcomes were heavily based on youth self-reports of learning, with the exception of the three focal youth for whom additional sources of data were collected. Despite the fact that interviews were in-depth, there is the possibility participants could not accurately recall the specificities of their interactions with nature, or their perceptions of learning outcomes have been flawed, either overstated or understated, depending on their level of confidence. Future studies should seek to combine self-reports with additional sources of data, such as performance tests, to assess knowledge and skills, track youth interactions (e.g. log files) on the iNaturalist website/app and evaluate how agency develops over time through follow-up longitudinal studies.

Also, participants were young people who could self-regulate and direct their learning (in the field and online) suggesting that they have considerable levels of autonomy and motivation to pursue and implement independent tasks with limited or no facilitation by others. These personality characteristics, alongside an a priori interest in science for some youth, may have had a positive impact on how they were engaged with tasks (attention and effort given to iNaturalist tasks) and associated learning outcomes. Learning outcomes may have been less prominent if youth without the above characteristics were studied such as young people who require ongoing support and scaffolding to implement a CCS task or those with no interest in science and/or the environment. Future studies should seek to engage and study more diverse young people in terms of interest in science and engagement with learning to identify how hybrid biodiversity activities should be designed to cater for their needs.

Lastly, given the limited previous research examining young people and their participation in CCS programmes, it becomes difficult to decipher whether the sample of youth examined in this study, which was self-selected, reflects the young people who are likely to take part in CCS programmes. This is applicable to both the interview data and the production of the three vignettes, the latter selected based on whether multiple data sources for individual youth were available. Our analysis showed that youth have varied prior science experiences (from limited to significant), suggesting that CCS comprises an entry point to engaging with science or a means to pursue existing science interests further. As a future direction, it would be useful if CCS providers capture and make public the demographics of CCS youth, as a baseline to comparing youth study samples with.

## Conclusions

Youth participation in authentic or real science is an effective approach to science teaching and learning. In this study, young people including those with limited prior science experiences were found to direct their participation and learning in hybrid CCS in ways that enabled specific forms of science learning as captured by the Environmental Science Agency framework. The *blended learning framework for biodiversity monitoring,* introduced in this paper, suggests that *settings, tools, activities* and *scaffolding* are required components to designing and implementing an educational, hybrid, biodiversity programme that can lead to specific *learning processes and outcomes*. Mobile biodiversity applications, as tools used in the field, were found to enhance and augment engagement with nature while also promoting learning. Scaffolding enabled by the design of technology, interactions with others and access to external resources is a key aspect of a successful learning experience. The mapping of participation types to specific learning outcomes gives directions of how CCS programmes should be designed to promote certain forms of learning.

The overall design of iNaturalist focuses on specific steps of the scientific process, specifically collecting and analysing (identifying) data by making species observations. These affordances could explain why learning outcomes such as understanding the norms of science and displaying scientific thinking were less evident in our analysis, as iNaturalist itself does not introduce users to these aspects. In contrast, learning outcomes related to the process of data collection such as knowledge and confidence in using the tools were much more prominent in our dataset. Intended learning outcomes should be made explicit and used to inform the design of hybrid CCS platforms such as iNaturalist and associated field-based programmes. For example, engagement with scientists and facilitators could aim to teach young people some of the norms of science such as how best to observe and find species and how to take a photograph that can be easily identified by others.
